# In vitro detection of circulating tumor cells compared by the CytoTrack and CellSearch methods

**DOI:** 10.1007/s13277-015-3105-z

**Published:** 2015-01-22

**Authors:** Thore Hillig, Peer Horn, Ann-Britt Nygaard, Anastasiya S. Haugaard, Sarah Nejlund, Ivan Brandslund, György Sölétormos

**Affiliations:** 1 Department of Clinical Biochemistry, Nordsjællands Hospital, Hillerød, University of Copenhagen, 3400 Hillerød, Denmark; 2 Department of Clinical Immunology and Biochemistry, Vejle Sygehus, 7100 Vejle, Denmark

**Keywords:** CTC, Recovery, Enumeration, CytoTrack, CellSearch

## Abstract

Comparison of two methods to detect circulating tumor cells (CTC) CytoTrack and CellSearch through recovery of MCF-7 breast cancer cells, spiked into blood collected from healthy donors. Spiking of a fixed number of EpCAM and pan-cytokeratin positive MCF-7 cells into 7.5 mL donor blood was performed by FACSAria flow sorting. The samples were shipped to either CytoTrack or CellSearch research facilities within 48 h, where evaluation of MCF-7 recovery was performed. CytoTrack and CellSearch analyses were performed simultaneously. Recoveries of MCF-7 single cells, cells in clusters, and clusters were determined. The average numbers of MCF-7 cells/cells in clusters/clusters recovered from blood by the CytoTrack and CellSearch methods were 103 ± 5.9/27 ± 7.9/11 ± 3.5 (95 % CI) and 107 ± 4.4/20 ± 7.1/10 ± 3.5, respectively, with no difference between the two methods (*p* = 0.37/*p* = 0.23/*p* = 0.09). Overall, the recovery of CytoTrack and CellSearch was 68.8 ± 3.9 %/71.1 ± 2.9 %, respectively (*p* = 0.58). In spite of different methodologies, CytoTrack and CellSearch found similar number of CTCs, when spiking was performed with the EpCAM and pan cytokeratin-positive cell line MCF-7. The results suggest that CytoTrack and CellSearch have similar abilities to identify CTC in vitro.

## Introduction

Cancer is a disease, which frequently have serious consequences. Cancer dissemination from the primary tumor to lymph nodes, distant organs, or bones leads to a poorer prognosis. Cancer may spread through invasion of the adjacent tissue or the lymphatic system, or through the blood circulation [1]. Subsequently, there can be circulating cancer cells in the blood stream of a cancer patient. These cells are known as circulating tumor cells (CTC), and it is possible to isolate, enumerate, and characterize them based on their molecular and genetic characteristics. Normally, the CTC will not be detected in the peripheral blood of a healthy individual. However, CTC detection may be indicative of cancer dissemination and can consequently be used for cancer prognosis. Therefore, CTC are potential biomarker for the surveillance of cancer progression [2, 3].

The detection of CTC in cancer patients has been pioneered by the CellSearch^™^ system, with numerous clinical studies that show a strong prognostic value of CTC detection in patients. The presence of CTC confers a poorer prognosis for the patient and shorter progression free (PFS) and overall survival (OS) in breast cancer [4] and ovarian cancer [5] as well as a shorter overall survival in colorectal cancer [6] and prostate cancer [7]. The collection of a CTC sample can be performed at any time before or during therapy and indicates subsequent rapid progression and mortality of the patients [8]. This has spurred several prospective clinical trials that examine the utility of CTC analysis in patient management. STIC CTC METABREAST is a two-armed trial comparing conventional treatment versus CTC-driven treatment, where patients with CTCs in the circulation receive chemotherapy as compared to hormone therapy in the CTC-negative patients. In the SWOG0500 trial, patients positive for CTC both before and after the first two cycles of chemotherapy are being treated by either continued chemotherapy (standard arm) or an alternative chemotherapy regimen to be decided by the oncologist (CTC arm). In the CirCe01 trial, patients positive for CTC after first cycle of new chemotherapy are shifted to next line therapy (CTC arm). In the treat CTC trial, patients with non-metastatic disease and the presence of HER2 non-amplified primary tumors that have at least one CTC by analysis of 15 mL of blood (two tubes) are treated with HER2 antibody therapy (Trastuzumab, CTC arm). In the DETECT III trial, patients with one or more CTC positive for HER2 are treated by the HER2 and EGFR tyrosine kinase inhibitor Lapatinib (CTC arm) [3]. In addition, a range of studies includes CTC retrospectively as a prognostic marker or a marker for monitoring treatment effect, with 1535 completed or ongoing studies including CTC registered on Clinicaltrials.org (2nd of July 2014).

The clinical application of CTC enumeration and characterization is thus under investigation and most studies are performed with the CellSearch method. However, the chosen methodology for CTC detection can have a major impact on the result, depending on sensitivity, specificity, and detection limit, and therefore the different methodologies should be compared with respect to their performance [2].

In the current study, the CTC detection methods CytoTrack^™^ and CellSearch are compared with an in vitro assessment using spiking experiments with the breast cancer cell line MCF-7 positive for both epithelial cell adhesion molecule (EpCAM) and pancytokeratin. The MCF-7 cell line was selected to avoid differences due to antibody specificities when comparing the two technologies.

## Methods and materials

Cells were cultured and spiked into blood from healthy donors after receiving written consent in accordance with the Declaration of Helsinki and approved by the national Danish Ethics Committee (protocol no. H-4-2014-056) for analysis by either CellSearch (Janssen Diagnostics, LLC, Raritan, NJ, USA formerly Veridex) or by CytoTrack (CytoTrack ApS, Lyngby, Denmark) after 48 h as outlined below.

### Cell culture

The human breast adenocarcinoma cell line MCF-7 was obtained from American Type Culture Collection (ATCC, Manassas, VA, USA) and cultured in Dulbecco’s Modified Eagle (DMEM)-GlutaMAX^™^ medium (Gibco, Grand Island, NY, USA), supplemented with 1 % penicillin-streptomycin and 10 % heat-inactivated fetal calf serum. The culture was grown in cell culture flasks in a humidified atmosphere containing 5 % CO_2_ at 37 °C. Cell culture was washed with Dulbecco’s phosphate buffered saline without calcium and magnesium (DPBS, Gibco) followed by harvesting with TrypLe Express (Gibco).

### Blood sample collection

Peripheral blood (7.5 mL) was collected from healthy donors into evacuated CellSave^™^ Preservative Tubes (Veridex LLC, Raritan, NJ, USA) containing EDTA and a cellular preservative. Blood samples were maintained at room temperature and spiked within 48 h.

### Blood spiking experiments

Six separate experiments were performed spiking five 7.5 mL blood samples with 150 MCF-7 cells as previously described [9] and analyzed by either CytoTrack or CellSearch. Additionally, a control sample was included in every experiment.

### CytoTrack

#### Sample processing

Spiked blood samples were centrifuged at 2500*g* for 15 min and the layer with leukocytes and tumor cells was transferred to 15 mL conical Falcon tubes (VWR Bie & Berntsen, Soeborg, Denmark). Remaining red blood cells were lysed with FACS lysing solution (BD Biosciences) and the samples centrifuged at 2500*g* for 15 min. Thereafter, cells were stained using CTC Stain^™^ comprising a mixture of anti-CD45/Near InfraRed (NIR) antibody (clone: HI30), anti-pancytokeratin/Green antibody (clone: AE1/AE3), 4′,6-diamidino-2-phenylindole (DAPI), and a permeabilization buffer (Intrastain, DAKO, Glostrup Denmark). Cells were then washed with phosphate buffered saline (PBS) with 1 % BSA and resuspended in H_2_O, transferred to a CytoDisc^™^, air-dried, and mounted using VectaShield Hard Set Mounting Medium (Vector Laboratories Inc., Burlingame, CA, USA) and covered with a CytoCover^™^.

#### Analysis

**Fig. 1:**
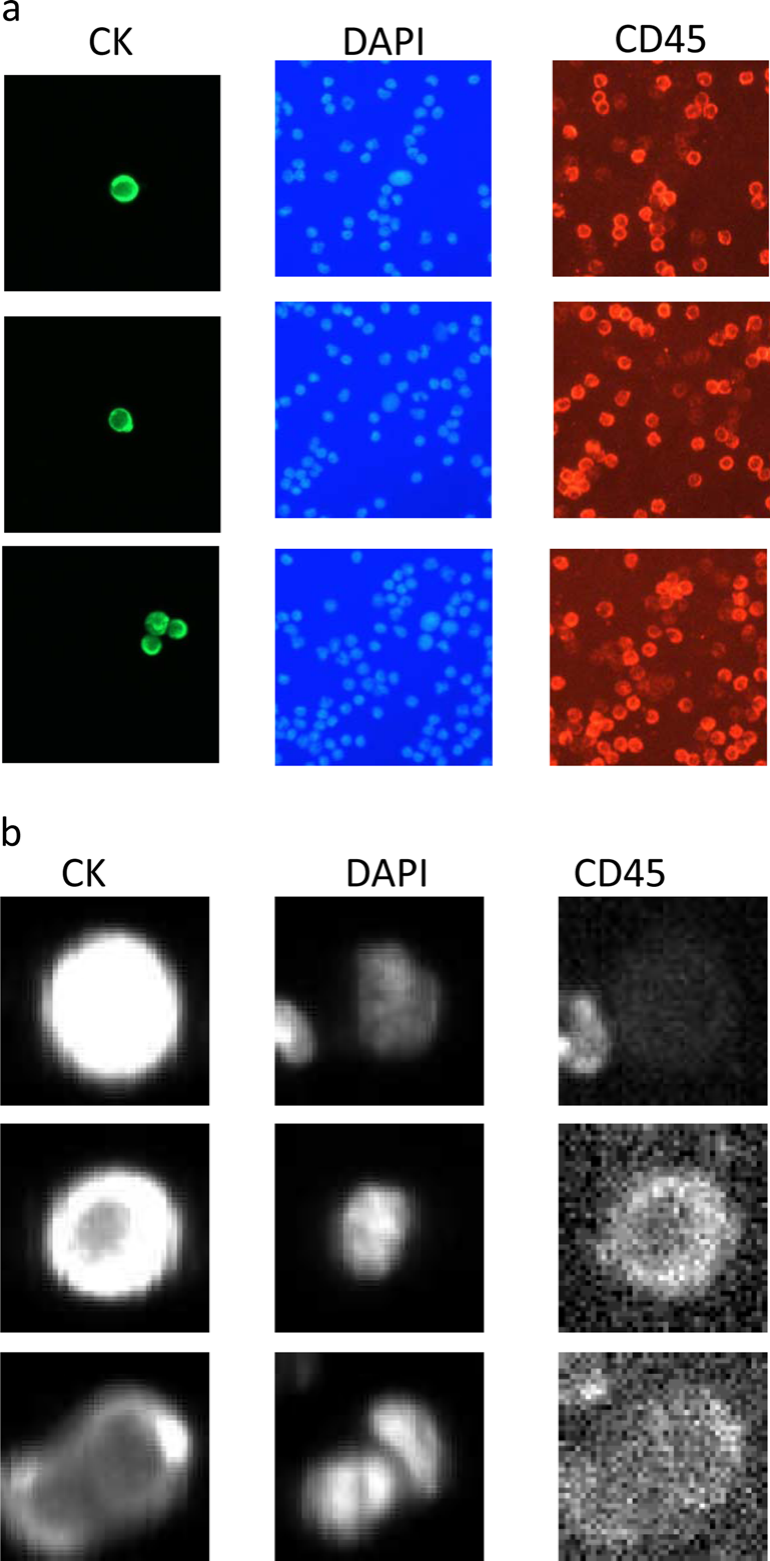
Image galleries of **a** CytoTrack and **b** CellSearch. Cells are stained with the epithelial marker pan-cytokeratin (left), the nuclear marker DAPI (center), and the leukocyte marker CD45 (right). CytoTrack images were recorded with a color camera, and CellSearch images were recorded with a black and white camera

Stained cells on the CytoDisc were enumerated by scanning using a CytoTrack CT4 Scanner. Scanning was performed within 1 week as previously described [9]. Briefly, green fluorescence events were recorded and listed in a hotspot table. Recorded events were visually inspected by the operator in the green channel, and an image gallery was automatically generated using the DAPI, green, and NIR channels from positions on the CytoDisc with suspected CTC (Fig. 1a). The image gallery was analyzed using the following morphologic criteria to identify CTC: Nearly round and size >4 μm with visible DAPI-positive nucleus with at least 50 % association with the cytoplasm, cytokeratin positive, and CD45 negative. The definition of CTC in the current study is similar to the definition used by other methods analyzing for CTC [2, 10–12].

### CellSearch

#### Sample processing

CellSearch CTC analysis was carried out on the CellSearch System consisting of the Autoprep^™^ and Celltracks Analyzer II^™^ systems. Suspected CTC were quantified using CellSearch Epithelial Cell Kit, ref 7900000, according to the manufacturer’s instructions. In short, 7.5 mL of whole blood was mixed with 6 mL of buffer and centrifuged at 800*g* for 10 min before placement in the semi-automated Autoprep system. Plasma was aspirated and suspected CTC of epithelial origin were immune-magnetically enriched using ferrofluid-conjugated antibodies specific for EpCAM. For identification of CTC permeabilization buffer, phycoerythrin (PE) conjugated antibodies specific for cytokeratins 8, 18, and 19 and DAPI for associated nuclei stain were added to the enriched cells. Residual leukocytes with positive DAPI were negatively differentiated from suspected CTC by addition of anti-CD45 antibodies conjugated with APC. After enrichment, staining, incubation, and washing, the Autoprep resuspended and transferred the cells to a magnetic cartridge that orient the ferrofluid bound cells in a monolayer focal plan for analysis in the Celltracks Analyzer II four color fluorescence microscope.

#### Analysis

Within 24 h, the magnetic cartridge was placed in the CellSearch Autoprep system and the semi-automated fluorescence microscope captured four color images (PE, DAPI, APC, and DiOC) of the entire magnetic cartridge focal plan. A software algorithm built an image gallery of objects positive for PE, cytokeratins, and DAPI nuclei stain (Fig. 1b). A single-trained operator makes the final selection of expected CTC from the image gallery to ensure that all CTC criteria stated in the *CellSearch* scanning paragraph were1 met.

### Statistical analysis

Data were tested by the D’Agostino-Pearson K2 test Gaussian distribution [13] and were not proven different from a Gaussian distribution. Mean, SD, 95 % confidence intervals (95 % CI), and percent coefficient of variation (CV%) were calculated using Excel (Microsoft, Redmond, Washington, USA). The two methods were compared pairwise and with regard to intra- and inter-serial runs by two-tailed *t* test with a significance level of *p* < 0.05.

## Results

**Fig. 2:**
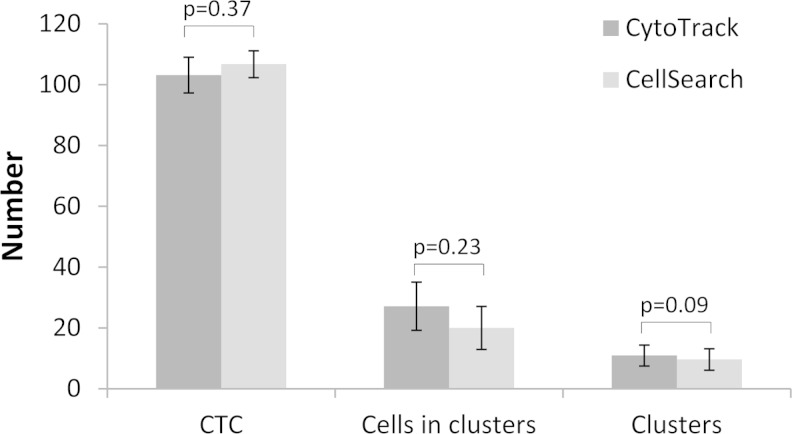
Number of CTC, cells in clusters, and clusters found with CytoTrack and CellSearch. Numbers are means of three spiking experiments to a total of 15 samples (CytoTrack) and 14 samples (CellSearch)

Results from each experiment are shown in Table 1 and Fig. 2. The average numbers of MCF-7 cells/cells in clusters/clusters recovered from blood by the CytoTrack and CellSearch methods were 103 ± 5.9/27 ± 7.9/11 ± 3.5 (95 % CI) and 107 ± 4.4/20 ± 7.1/10 ± 3.5 (95 % CI), respectively, with no difference between the two methods (*p* = 0.37/*p* = 0.23/*p* = 0.09). Overall, the recovery of MCF-7 cells by CytoTrack and CellSearch was 68.8 ± 3.9 %/71.1 ± 2.9 %, respectively (*p* = 0.58). Overall, the intra assay variability of CytoTrack and CellSearch was 11.2 % (range 11.3–12.5 %) and 7.9 % (range 3.2–7.9 %), respectively. A test for intra assay variability for CytoTrack and CellSearch showed no difference between the three spiking experiments (*p* > 0.05, data not shown) in either method.

**Table 1:** Number of CTC found in spiking experiments with CytoTrack and CellSearch

	CytoTrack				CellSearch			
CTC^1^	Exp. 1	Exp. 2	Exp. 3	Total	Exp. 1	Exp. 2	Exp. 3	Total
Mean	104	104	101	103	116	102	104	107
Range	93–122	88–123	89–122	88–123	107–122	98–107	95–117	95–122
n	5	5	5	15	4^2^	5	5	14
95 % CI	10,3	11,1	11,0	5,9	6,4	2,9	7,2	4,4
Recovery %	69,2	70,0	67,1	68,8	77,3	68,1	69,2	71,1
CV	11.3 %	12.1 %	12.5 %	11.2 %	5.6 %	3.2 %	7.9 %	7.9 %

^1^CTC are defined as nearly round size >4 μm with visible DAPI-positive nucleus with at least 50 % association with the cytoplasm, pancytokeratin positive, and CD45 negative

^2^One sample failed in CellSearch Exp.1

## Discussion

The analysis of CTC with high sensitivity and specificity is challenging, as it is required to pinpoint individual CTC in a 7.5 mL blood sample with more than 50 million leukocytes [14]. In the current study, the two technologies CytoTrack and CellSearch are compared under in vitro conditions. CytoTrack is a novel method recently described [9] and the CellSearch CTC Test is FDA-approved as an aid in the monitoring of patients with metastatic breast, colon, and prostatic cancer [15]. It should be pointed out that the CytoTrack technology has as yet not been clinically validated although work is ongoing with serial measurements of CTC among patients with metastatic breast cancer [16]. The two technologies use very different approaches. Whereas the CellSearch approach is a ‘closed’ automated, FDA cleared system intended for clinical monitoring, the CytoTrack approach is an ‘open’ manual research system which allow for studying other markers for CTC enumeration and characterization of CTC. In CytoTrack, all nucleated cells in a blood sample are DAPI-stained, immunostained with antibodies against cytokeratin and CD45 and cytosmeared over a CytoDisc. Afterwards, fluorescence scanning is performed, suspected CTC on the disc are identified, and an image gallery is created for manual validation. In CellSearch, plasma is removed and magnetic sorting of EpCAM positive cells is carried out, subsequently the cells are stained for cytokeratins 8, 18, and 19, DAPI and CD45 and placed in a magnetic cartridge where image galleries of suspected CTC events are recorded for manual validation. The different approaches could in principle give rise to significantly different abilities to detect CTC, with CytoTrack relying on cytokeratin signal to detect cells and CellSearch dependent on both EpCAM and cytokeratin expression. In the current study of in vitro detection of MCF-7, a cytokeratin- and EpCAM-positive cell line, the two CTC technologies CytoTrack and CellSearch have similar recovery of cells spiked into blood (69 vs. 71 %, *p* = 0.58). It should be noted that the recoveries were estimated on the basis of FACSAria spiking counts of cells in blood samples, a count which was previously estimated to result in actual spiking numbers around 74 % of the expected FACSAria spiking cell counts [9]. The spiking was performed with care taken to obtain single cells; however, a fraction of cell clusters contained two or more cells. Therefore, the results of both CTC number (total number of both single cells and cells in clusters), cells in clusters (only cells in clusters counted), and clusters (the number of clusters counted) were recorded. Both CTC, cells in clusters and clusters had similar recoveries by CytoTrack and CellSearch. CellSearch, however, has a lower variability (CV%) in the analysis, which may be due to the automated procedures in CellSearch versus manual procedures in the CytoTrack analysis.

Nonetheless, CytoTrack proves to have a recovery and a reproducibility matching that of CellSearch, which is promising with regard to future studies including test of CTC in clinical samples from breast, colon, and prostate cancer patients. Moreover, further markers, both surface and intracellular phenotypic and genetic, can be explored to improve the performance of the CytoTrack across different cell types. A specific challenge for CellSearch is when CTC undergo epithelial to mesenchymal transition (EMT) as the EpCAM expression is reduced or not present in mesenchymal cells which makes it difficult to detect these cells [17, 18]. The possibility to explore markers in cells of mesenchymal origin thus seems especially interesting since if markers for EMT are present on the surface or inside CTC it is possible for CytoTrack to find the cells. This is the first in vitro comparison between CytoTrack and CellSearch, and considerable work is required for further comparison. CytoTrack is a novel technology and thus there is a need for more in vitro studies of cells from different cancer types spiked into blood and in vitro studies with EpCAM or CK weak cells to investigate the limits of the two technologies. Also, studies of basic clinical performance in various cancers as well as studies of intra- and inter-lab and inter-observer variability are required. In conclusion, our data indicate that CytoTrack and CellSearch despite fundamentally different technologies may yield similar results when using a cell line selected to match the markers used by both technologies. Our investigation provides a basis for further studies using either different cells lines and/or different CTC markers.
